# Investigation of Low‐Current Direct Stimulation for Rehabilitation Treatment Related to Muscle Function Loss Using Self‐Powered TENG System

**DOI:** 10.1002/advs.201900149

**Published:** 2019-06-12

**Authors:** Jiahui Wang, Hao Wang, Tianyiyi He, Borong He, Nitish V. Thakor, Chengkuo Lee

**Affiliations:** ^1^ Department of Electrical and Computer Engineering National University of Singapore 4 Engineering Drive 3 117576 Singapore; ^2^ Singapore Institute for Neurotechnology (SINAPSE) National University of Singapore 28 Medical Drive, #05‐COR 117456 Singapore; ^3^ Hybrid‐Integrated Flexible (Stretchable) Electronic Systems Program National University of Singapore 5 Engineering Drive 1 117608 Singapore; ^4^ NUS Suzhou Research Institute (NUSRI) Suzhou Industrial Park, Suzhou 215123 P. R. China

**Keywords:** electrical muscle stimulation, self‐powered, stimulation efficiency, stimulation stability, stimulation waveform, triboelectric direct stimulation

## Abstract

Muscle function loss is characterized as abnormal or completely lost muscle capabilities, and it can result from neurological disorders or nerve injuries. The currently available clinical treatment is to electrically stimulate the diseased muscles. Here, a self‐powered system of a stacked‐layer triboelectric nanogenerator (TENG) and a multiple‐channel epimysial electrode to directly stimulate muscles is demonstrated. Then, the two challenges regarding direct TENG muscle stimulation are further investigated. For the first challenge of improving low‐current TENG stimulation efficiency, it is found that the optimum stimulation efficiency can be achieved by conducting a systematic mapping with a multiple‐channel epimysial electrode. The second challenge is TENG stimulation stability. It is found that the force output generated by TENGs is more stable than using the conventional square wave stimulation and enveloped high frequency stimulation. With modelling and in vivo measurements, it is confirmed that the two factors that account for the stable stimulation using TENGs are the long pulse duration and low current amplitude. The current waveform of TENGs can effectively avoid synchronous motoneuron recruitment at the two stimulation electrodes to reduce force fluctuation. Here, after investigating these two challenges, it is believed that TENG direct muscle stimulation could be used for rehabilitative and therapeutic purpose of muscle function loss treatment.

## 1 Introduction

In recent years, implantable biomedical devices have progressed rapidly. Various implantable devices are developed for healthcare monitoring purpose, such as chemical sensors to detect glucose and lactate,^1^, ^2^ flexible electronics to record electrophysiological signals,^3^ optoelectronic photometers to monitor neuronal dynamics,^4^ and pressure sensors for blood flow monitoring^5^ and orthopedic applications.^6^ Apart from healthcare monitoring, implantable biomedical devices are also widely employed to deliver therapeutic interventions. Examples include optoelectronic devices for neuromodulation,^7^ soft mechanical actuators to assist organ functions,^8^, ^9^ and microfluidic devices for drug delivery.^10^, ^11^ Despite these emerging therapeutic interventions, electrical stimulation remains as a dominating therapeutic method. To interface with the delicate biological tissues, flexible electrodes made of polyimide,^12^, ^13^, ^14^ SU‐8,^15^, ^16^ poly(lactic‐*co*‐glycolic acid) (PLGA),^17^ and hydrogel^18^ are developed to minimize the mechanical mismatch with the brain and peripheral nerves. Meanwhile, high‐density silicon‐based microelectrode arrays, such as the Neuropixels probe^19^ and Utah electrode array,^20^ also show huge clinical potentials.

In human body, electrical signals carry neuronal information and regulate neuronal activities. Electrical stimulation has been widely applied to treat neurological disorders and to recover lost functions caused by nerve injuries, such as deep brain stimulation to relieve Parkinson's disease,^21^, ^22^ cochlear nerve stimulation to restore acoustic hearing,^23^ spinal cord stimulation to augment recovery and restore limb functions,^24^, ^25^, ^26^ and peripheral nerve stimulation to assist bladder functions.^27^ Neurological disorders and nerve injuries can lead to muscle function loss, including stroke and spinal cord injury in the central nervous system, and neuropathy in the peripheral nervous system. For muscle function loss, initial symptoms include increasing muscle atrophy^28^ and may deteriorate to paralysis,^29^ in which case, muscle function is completely lost. Electrical muscle stimulation has been clinically applied to prevent^30^ and reverse^31^ muscle disuse atrophy, and to recover meaningful muscle movements.^32^ In terms of the hardware for electrical muscle stimulation, waveform generators and power supply are important components.

**Figure 1:**
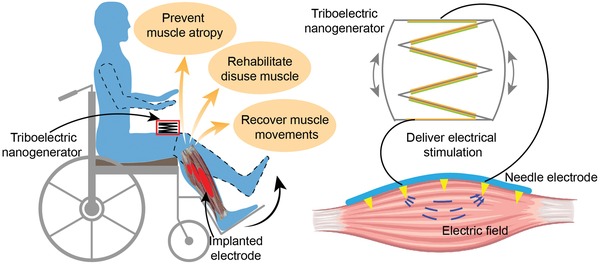
Illustration of electrical muscle stimulation directly powered by TENG. Electrical pulses generated by the TENG are connected to the penetrating electrodes to directly stimulate muscle.

While self‐powered triboelectric nanogenerator (TENG) sensors made significant impact to wearable electronics ranging from healthcare, human–machine interface, robotics, gaming, virtual reality, and augmented reality,^33^, ^34^, ^35^, ^36^, ^37^, ^38^, ^39^, ^40^, ^41^, ^42^ TENG also shows great potential to serve as both waveform generator and power supply in the implantable electrical muscle stimulation systems, considering the recent successful demonstrations of direct electrical stimulation on the cells, peripheral nerves, and brain using current output from the TENGs. On the cell level, Guo et al. showed electrical stimulation using TENG output enhances neural differentiation,^43^ and Long et al. studied wound healing and antibiofouling using the electric field generated by TENGs.^44^, ^45^ On the peripheral nerve level, Zhang et al. first demonstrated direct TENG stimulation on the frog's sciatic nerve.^46^ Then, Lee et al. further investigated the high stimulation efficiency of TENG on rat's sciatic nerve and pelvic nerve.^47^, ^48^ Furthermore, Yao et al. studied weight control by stimulating rat's vagus nerve powered by TENGs.^49^ On the brain level, Fu et al. and Dai et al. further demonstrated direct TENG stimulation on rat's somatosensory cortex and motor cortex.^50^, ^51^ The TENGs work as ion pumps when energy conversion is achieved by coupling between the triboelectric effect and the electrostatic effect, and TENGs can be potentially developed into fully implantable electrical muscle stimulation systems to replace the currently available wearable TENG solution as demonstrated in the previous peripheral nerve and brain stimulation works, and such TENGs can simultaneously serve as waveform generator and power supply to provide therapeutic interventions to the muscles through implanted electrodes (**Figure** **1**).

Although the pioneering works have demonstrated successful direct TENG stimulation on the peripheral nerves and brain, direct TENG muscle stimulation faces new challenges. Different from the peripheral nerves and brain which are full of excitable neurons and have low stimulation threshold, muscles are constituted of motor units, where distributed motoneurons are electrically activated to drive the muscle fibers they innervate and typically require stimulation current of mA‐level.^31^, ^52^ Thus, the first challenge of direct TENG muscle stimulation is to achieve high stimulation efficiency to enable muscle activation with µA‐level TENG output. Furthermore, compared to voluntarily controlled muscle movements, electrical stimulation causes rapid force output change, which is characterized as decrease in force‐generating capability of muscle.^53^, ^54^, ^55^, ^56^ Such force output change affects precise muscle modulation using conventional square wave stimulation, but its neural basis is not well understood. The reason for increased fatigability using electrical stimulation has been thought to be twofold. On the one hand, electrical stimulation preferably recruits large‐diameter motoneurons at lower input current amplitude, which is opposite to the normal physiological order.^57^, ^58^ On the other hand, electrical stimulation tends to recruit all motoneurons simultaneously.^59^, ^60^ Closed‐loop stimulation systems are used to compensate the changing force and achieve stable force output.^61^, ^62^, ^63^ Looking further into the future applications, if TENG activates muscle in the same mechanism as the conventional square wave stimulation, stable force output for precise muscle modulation may become the second challenge of direct TENG muscle stimulation.

Here, we investigate the above two challenges regarding direct TENG muscle stimulation, using a stacked‐layer TENG to stimulate rat's tibias anterior (TA) muscle through a multiple‐channel epimysial electrode with penetrating spikes (Figure 1). For the first stimulation efficiency challenge, we demonstrate direct TA muscle activation by the output of the stack‐layer TENG, and we further explore optimization of stimulation efficiency by changing electrode configurations of different spatial position. For the second stimulation stability challenge, we find the long‐pulse‐width, low‐current‐amplitude TENG stimulation show better force output stability, and we further validate the mechanisms of unstable force output during electrical stimulation using conventional square wave stimulation and enveloped high frequency stimulation.

## 2 Results and Discussion

### 2.1 System, Device Configuration, Benchtop Measurement, and In Vivo Testing Setup

**Figure 2:**
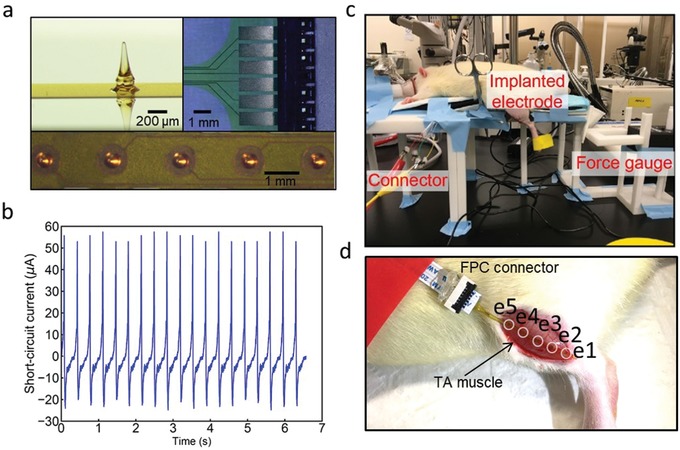
Experimental setup, device, and benchtop characterization. a) Photos of the penetrating electrodes and flexible printed circuit (FPC) connector. b) Short‐circuit current characterization of the TENG. c) Photo of the in vivo experimental setup. d) In vivo implantation of the penetrating electrodes on the TA muscle, where e1–e5 are the five electrode sites.

To access the widely distributed motoneurons in the muscle tissue, we used a multiple‐channel epimysial electrode. This spiked epimysial electrode is fabricated using microelectromechanical systems process^64^ and has five channels, formed by 3D SU‐8 electrodes coated with gold (**Figure** **2**a). Unlike the bulky conventional epimysial electrodes^65^, ^66^ which are just attached to the muscle surface, our compact epimysial electrode adheres well to the muscle with all the 3D SU‐8 electrodes penetrating into the muscle tissue. During muscle contractions, the conventional epimysial electrodes are vulnerable to detachment from muscle surface. However, our spiked epimysial electrode can effectively avoid this issue, as the penetrating electrodes remain in the muscle tissue and are tightly surrounded by muscle fibers to accommodate to muscle structure change during contractions. Such capability to remain good contact with muscle tissue ensures the stable electrode‐tissue interface impedance, which is of crucial importance to stably deliver electrical stimulation to the muscle tissue.

Here, we used a stacked‐layer TENG together with the spiked epimysial electrode as a prototype system to investigate the grand challenges in TENG direct muscle stimulation. The stacked‐layer TENG deforms with applied pressure to turn mechanical energy into electrical energy. The detailed fabrication process can be found in Figure S1 in the Supporting Information. To enhance the current output of the TENG device, triboelectric energy harvesters are connected in parallel structure. In electrical stimulation, current‐controlled waveform generator is generally preferred as compared to voltage‐controlled waveform generator, as the electrode‐tissue interface impedance may not remain constant. When electrode‐tissue interface impedance fluctuates, the voltage drop on this electrode‐tissue interface also shifts. If a voltage‐controlled waveform generator is used, then the effective voltage applied on the tissue is unstable. Considering that current‐controlled waveform is more stable, we characterized the current delivery capability of the stacked‐layer TENG. Since the inner impedance of the TENGs falls in several MΩ (5 MΩ for the stacked‐layer TENG, as shown in Figure S2, Supporting Information) and the tissue impedance is only around several kΩ, the short‐circuit current output of 75 µA (Figure 2b) well characterizes the stacked‐layer TENG current delivery capability for in vivo applications.

In vivo testing setup is shown in Figure 2c, and the detail description can be found in the Experimental Section. We use the TA muscle as a demonstration here, as the activation of TA muscle induces leg forward kicking movement and the generated force output can be measured with a force gauge. The zoomed‐in Figure 2d shows the spiked epimysial electrode implanted on the TA muscle surface, with all the five penetrating SU‐8 electrodes in the muscle.

### 2.2 In Vivo Mapping of TENG Stimulation Efficiency

**Figure 3:**
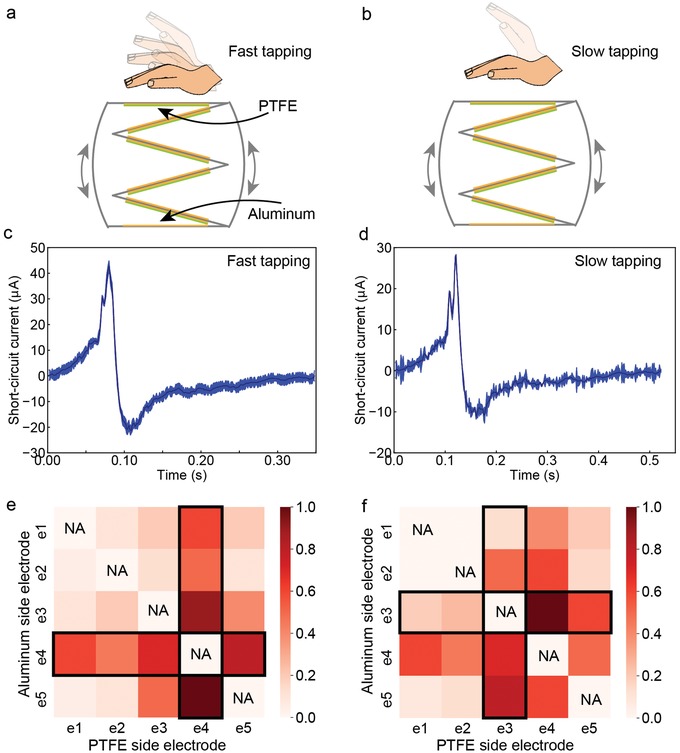
In vivo mapping of stimulation efficiency using different electrode configuration. a) Illustration of fast tapping. b) Illustration of slow tapping. c) Short‐circuit current waveform during fast tapping. d) Short‐circuit current waveform during slow tapping. e) Heatmap of stimulation efficiency under fast tapping. f) Heatmap of stimulation efficiency under slow tapping.

The working mechanism of TENGs relies on energy conversion by coupling between the triboelectric effect and the electrostatic effect, and thus the TENGs act as charge pumps, in which current flows back and forth between the two electrodes in alternating current (AC) characteristics.^67^ Here, we used polytetrafluoroethylene (PTFE) and aluminum as the two electrodes (**Figure** **3**a), and we define them as the PTFE side electrode and aluminum side electrode to facilitate the later discussion. To study TENG output waveforms, we applied fast tapping mode (Figure 3a) and slow tapping mode (Figure 3b) on the same stacked‐layer TENG. The short‐circuit current waveforms in correspondence with the two tapping mode are shown in Figure 3c,d, and they show difference in two aspects. In fast tapping mode, the short‐circuit current is of higher peak amplitude and shorter pulse duration. This can be easily understood from the working mechanism of the stacked‐layer TENGs. When the stacked‐layer TENG is pressed, layers of triboelectric energy harvesters contact with each other to generate triboelectric surface charge on PTFE and the aluminum film, and the resultant current flows through the electrode to stimulation muscle tissue. In fast tapping mode, the contact of triboelectric layers happens in a shorter duration, which results in a shorter current pulse duration. To keep the charge amount constant in different tapping mode, a shorter current pulse duration results in a higher peak amplitude.

During in vivo testing, we connected any two electrode sites on the epimysial electrode to the two sides of the stacked‐layer TENG, as PTFE side electrode and aluminum side electrode. To access the widely distributed motoneurons in the muscle tissue, we measured the TENG force output using different electrode configurations. For each tapping mode, the force output mapping results are summarized into a heatmap, as shown in Figure 3e,f. In this heatmap, the *x*‐axis represents the electrode site working as the PTFE side electrode and the *y*‐axis represents the electrode site working as the aluminum side electrode. The stimulation efficiency is calculated as the normalized force output (dividing each force output with the maximum force output achieved using this tapping mode) and is coded in color.

We have three observations in the stimulation efficiency heatmaps. First, stimulation efficiency differs when different electrode sites are used. In Figure 3e, the stimulation efficiency obtained by e1 as PTFE side electrode and e2 as aluminum side electrode is much lower than the stimulation efficiency when using e4 as PTFE side electrode and e5 as aluminum electrode. In addition, the heatmap shows higher stimulation efficiency when certain electrode sites are involved in the electrode configurations. For example, in Figure 3e, when e4 is involved, either as the PTFE side electrode or the aluminum side electrode, the stimulation efficiency is higher. Such correlation between a certain stimulation electrode site and the stimulation efficiency indicates motoneuron spatial distribution pattern, as when the electrode site is close to the motoneurons, stimulation efficiency gets improved. Second, when the PTFE and aluminum side electrodes are switched, stimulation efficiency does not remain the same, which is characterized as the asymmetrical heatmap pattern. For instance, in Figure 3e, stimulation efficiency obtained by using e4 as PTFE side electrode and e5 as aluminum side electrode is higher than the stimulation efficiency when using e5 as PTFE side electrode and e4 as aluminum side electrode. The asymmetrical heatmap pattern is due to the asymmetrical positive and negative current peaks generated in one cycle of the stacked‐layer TENG pressing and releasing, as shown in Figure 3c,d. Third, the heatmap changes in correspondence with different current waveforms generated by fast and slow tapping mode. In fast tapping mode, e4 is involved in the electrode configurations of the optimum stimulation efficiency. However, in slow tapping mode, electrode configurations involving e3 achieves the optimum stimulation efficiency.

Up to here, we have studied the first challenge concerning TENG muscle stimulation efficiency. We have demonstrated successful TENG direct muscle stimulation (Movie S1, Supporting Information). In addition, the heatmap results show the stimulation efficiency is affected by three factors, namely, the spatial location of the electrode sites, the electrode sites connection to the TENG, and the current waveforms generated by the TENG. Since in real applications, we want to employ the optimum stimulation efficiency, and systematic mapping needs to be done to find the electrode configurations for the optimum stimulation efficiency. Then, we will move on to the investigation of the second challenge regarding the stability of TENG muscle stimulation.

### 2.3 In Vivo Stability of Muscle Stimulation Induced by TENG Current Output, Conventional Square Wave Stimulation, and Enveloped High Frequency Stimulation

**Figure 4:**
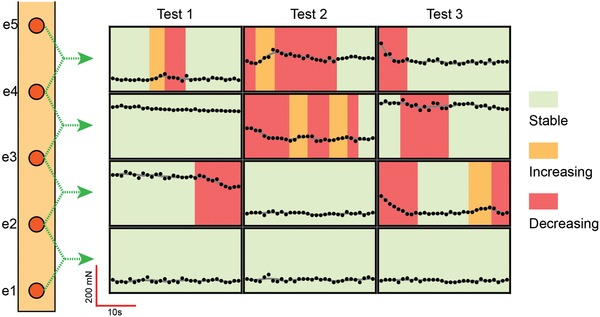
Force profiles measured with different electrode configuration powered by the TENG. For each electrode configuration, the electrode site with smaller numbering is used as PTFE side electrode. The force profile showing stable, increasing, and decreasing trends is marked in green, orange, and red, respectively.

To study force output stability of TENG muscle stimulation, we repeated the TENG muscle stimulation using different electrode configurations on three animals (**Figure** **4**). For each TENG stimulation, a pair of neighboring electrode sites was used, and the electrode site with smaller numbering was always chosen as the PTFE side electrode. Force profiles using different electrode configurations varied in terms of output force amplitude, which agrees with our conclusion drawn from the heatmaps in Figure 3, as different electrode sites have different distance to distributed motoneurons in the muscle tissue and have distinct stimulation efficiency. Furthermore, we marked the force profile with stable, increasing, and decreasing changing trends in different colors. For some measured force profiles, we observed fluctuations of increasing and decreasing trends. If the decreasing force profile is explained as muscle fatigue, then what will account for the increasing force profile?

To further study the output profile stability, we tested square wave stimulation and enveloped high frequency stimulation. We chose to test square wave stimulation because it is a widely applied waveform for muscle stimulation.^53^, ^54^, ^55^, ^56^ Since recently interference high frequency stimulation receives huge attention in neuromodulation,^68^ we also tested the enveloped high frequency stimulation. Here, we only needed single channel for testing, so the further validation of these two waveforms employed commercial stainless‐steel wires. To generate these two waveforms, a commercial high current stimulator was used. Details about the testing setup, stimulator, electrode implantation, and these two waveforms can be found in Figure S4 in the Supporting Information.

**Figure 5:**
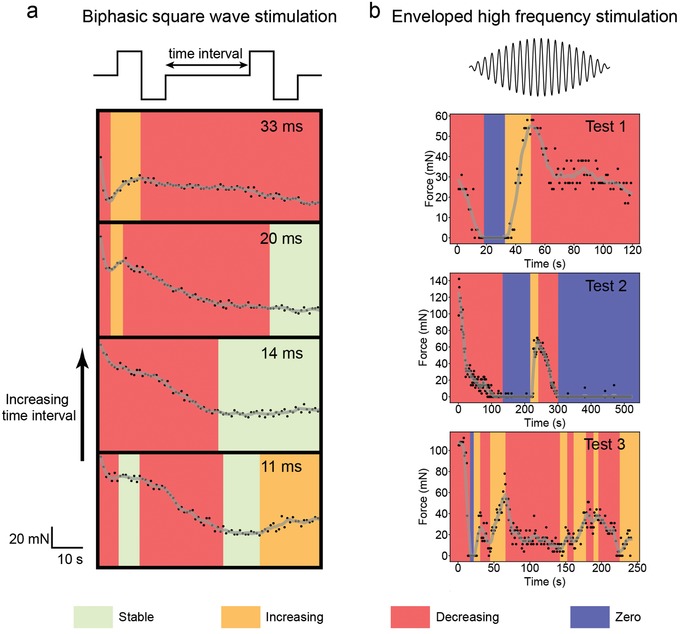
Unstable force profiles induced by a) square wave and b) enveloped frequency stimulation. The force profile showing stable, increasing, and decreasing trends is marked in green, orange, and red, respectively. In addition, the zero force output is marked in purple.

Force profiles for further validation of stimulation stability using square wave and enveloped high frequency stimulation are shown in **Figure** **5**. In line with the TENG force profiles (Figure 4), we also marked the force profiles with stable, increasing, and decreasing changing trends. In addition, we added color coding of zero output to mark the unique force profiles with enveloped high frequency stimulation. Here, we have three observations. First, same as the TENG force profiles, changing trends of increasing and decreasing occurred in square wave and enveloped high frequency stimulation as well. As compared to these two waveforms, TENG induced more stable force output. Second, muscle fatigue did exist in all testing, as force profiles tended to decrease in a long timescale. Third, enveloped high frequency stimulation induced very unstable force output. A unique and interesting phenomenon was the recovery of force output to even higher than the initial force amplitude, when the enveloped high frequency stimulation was continuously delivered to the muscle. Obviously, it could not be explained by muscle fatigue and it seemed like the state of the muscle tissue changed with the continuous presence of the enveloped high frequency stimulation. With the concept of muscle tissue state change, if we look back at the TENG and square wave force profiles, all the force fluctuations of increasing and decreasing in a short timescale can be easily understood. Since motoneurons are activated in electrical muscle stimulation, this muscle tissue state change is caused by motoneuron state change. We have a detailed explanation of motoneuron state change with the presence of electrical stimulation and it can be found in Figure S5 in the Supporting Information.

### 2.4 Modeling of Stimulation Waveform Influence on Motoneuron State Change

By comparing the force profiles in Figures 4 and 5, we have found that TENGs induce smaller motoneuron state change than the conventional square wave stimulation and enveloped high frequency stimulation, which manifests itself as less output force fluctuation. To investigate the factors that account for the more stable TENG muscle stimulation, we performed modeling to analyze the pulse width of TENG output, square wave stimulation, and enveloped high frequency stimulation, and the modeling circuit and parameters can be found in Figure S6 in the Supporting Information.

**Figure 6:**
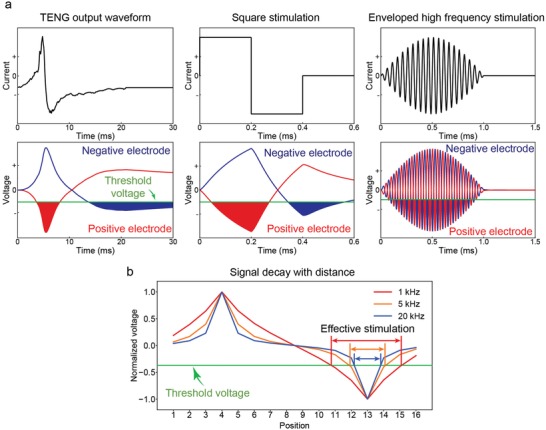
Modeling of how stability is affected by stimulation waveform frequency. a) Modeling of voltage waveform generated at the two stimulation electrodes. b) Stimulation of stimulation waveform and transmission distance. Stimulation of lower frequency transmits to further distance.

Comparison of voltage waveforms near the two stimulation electrodes when different current waveforms are applied reveals the first property of TENG stimulation: it avoids inducing synchronized neuron recruitment at the two stimulation electrodes (**Figure** **6**a). Due to the long pulse width of TENG output and square wave stimulation, voltage waveforms at the two stimulation electrodes exceed threshold voltage in different temporal patterns. However, for enveloped high frequency stimulation, voltage waveforms at the two stimulation electrodes exceed the threshold voltage in a highly synchronized pattern. The fast alternation of enveloped high frequency stimulation polarity leads to the synchronized temporal pattern for which the voltage waveforms at the two stimulation electrodes exceed threshold voltage.

The second property of TENG stimulation is that it recruits motoneurons located in a larger area around the stimulation electrodes (Figure 6b). Neural tissue has been found to be low‐pass filtering, allowing low frequency signal to propagate further.^69^, ^70^ Longer propagation distance of electrical stimulation means more motoneurons can be activated. Accordingly, we performed modeling on signal decay in neuromuscular tissue and confirmed that high frequency input current decays faster to only activate neurons located closer to the stimulation electrodes (Figure 6b). In frequency domain, the long pulse width TENG stimulation corresponds to low frequency stimulation and propagates further in neuromuscular tissue, to stimulate motoneurons distributed in a larger area.

Thus, combining these two properties, TENGs avoid synchronized recruitment of motoneurons located closely to the two stimulation electrodes in terms of both time and space. This asynchronous motoneuron recruitment is crucial to the observation of less force fluctuation with decrease and increase because it avoids motoneuron state shift in the same direction at the two stimulation electrodes (to increase or to decrease together). For TENG stimulation, motoneuron state shift varies at different location, so most of the time, the effect cancels each other, so that we only observed gradual force output fluctuation with TENG stimulation (Figure 4).

### 2.5 In Vivo Measurements: Stimulation of Lower Frequency and Smaller Amplitude Avoids Motoneuron Recruitment Synchronization

**Figure 7:**
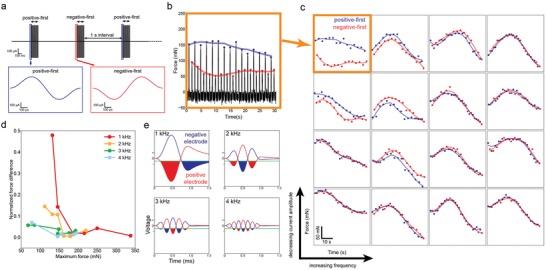
In vivo measurement of how stability is affected by stimulation waveform frequency and current amplitude. a) Positive‐first and negative‐first stimulation was alternatively delivered. b) A measured force profile. Force measured with positive‐first stimulation is marked in blue and force measured with negative‐first stimulation is marked in red. c) With lower frequency and lower amplitude, stimulation avoids synchronization of the motoneuron recruitment at the two stimulation electrodes (marked in orange).

The above modeling shows high frequency leads to synchronized motoneuron recruitment at the two stimulation electrodes. In addition, with increasing current amplitude, motoneuron recruitment at the two stimulation becomes saturated, and thus more synchronized. We further performed in vivo measurements to investigate the influence of frequency and amplitude on motoneuron recruitment at the two stimulation electrodes, using enveloped high frequency stimulation which amplifies motoneuron state change for clear validation. When positive‐first enveloped high frequency stimulation is delivered to neuromuscular tissue, motoneurons at the positive electrode experience positive‐first voltage waveform, while motoneurons at the negative electrode experience negative‐first voltage waveform. To access the synchronization of motoneuron recruitment at the two stimulation electrodes, we investigated how force output differs in response to positive‐first and negative‐first current input. To avoid accumulated muscle fatigue, positive‐first and negative‐first stimulation were alternatively applied (**Figure** **7**a). If motoneuron recruitment at the two stimulation electrodes was synchronized, a smooth force profile without abrupt change in between the prior and subsequent force output was expected. Otherwise, the prior force output was expected to be different from the subsequent position (Figure 7b).

Figure 7c shows the force profiles induced by alternating positive‐first and negative‐first stimulation. At low frequency and small amplitude, force output profile induced by positive‐first and negative‐first stimulation formed two well‐separated curves, which clearly showed the asynchronous neuron recruitment at the two stimulation electrodes. With increasing enveloped high frequency stimulation frequency and amplitude, the two well‐separated force output curves at low frequency and amplitude merged, where the force induced by positive‐first and negative‐first stimulation could no longer be differentiated. The quantized analysis of the force output difference induced by positive‐first and negative‐first stimulation also confirmed that higher frequency and larger amplitude of EHFS improved neuron recruitment synchronization at the two stimulation electrodes (Figure S7, Supporting Information).

## 3 Conclusion

In this study, we demonstrated direct TENG muscle stimulation using a self‐powered system of stacked‐layer TENG and multiple‐channel spiked epimysial electrode. More than feasibility demonstration, we further investigated two grand challenges regarding TENG muscle stimulation. For the first stimulation efficiency challenge, we found TENG stimulation efficiency is affected by electrode configurations and a mapping needs to be done to find the optimum electrode configuration. The second grand challenge is the stimulation stability. By comparing TENG stimulation with the conventional square wave stimulation and enveloped high frequency stimulation, we found TENG muscle stimulation generates more stable force output. The further modeling and in vivo measurement indicated that the long pulse width and low current amplitude of TENG output accounts for the less force fluctuation during TENG stimulation, because the motoneuron recruitment synchronization at the two stimulation electrodes is avoided. Our work validates the possibility of direct TENG muscle stimulation and provides useful guidance for practical applications by looking into the stimulation efficiency and stability challenge. With the successful achievement of stable TENG muscle stimulation and the rapid development of TENG materials and devices in research community, we believe fully an implantable TENG system could be employed for rehabilitation treatment of muscle function loss in future.

## 4 Experimental Section

*In Vivo Experiment*: All experiments were conducted according to protocols approved by the Institutional Animal Care and Use Committee at the National University of Singapore. Five Sprague‐Dawley rats (around 450 g) were used for the acute experiments. Anesthesia was induced with isoflurane (Aerrane, Baxter Healthcare Corp., USA). Carprofen (Rimadyl, Zoetis, Inc., USA) was injected for pain relief before surgery. After the rat was anesthetized, fur on the leg was gently removed by a shaver. Then, the skin was disinfected with 70% ethanol wipes, and an incision was made with a surgical blade to expose the TA muscle. The spiked multiple‐channel epimysial electrode was implanted on the muscle surface and fixed by suture wires. The anesthetized rat was fixed on a stand, and the ankle of left leg was connected to a dual‐range force sensor (Vernier, USA). This force sensor was connected to a laptop through a data acquisition (DAQ). LabView (National Instruments, USA) was used for on‐site result visualization during the measurements. After the measurements, MATLAB was used for data analysis.

## Conflict of Interest

The authors declare no conflict of interest.

## Supporting information

Supplementary

Supplementary
