# Supplementary material for: Investigation of Low‐Current Direct Stimulation for Rehabilitation Treatment Related to Muscle Function Loss Using Self‐Powered TENG System
Source: Adv Sci (Weinh). 2019 Jun 12;6(14):1900149. doi: 10.1002/advs.201900149 (PMC6662055; doi:10.1002/advs.201900149)
Supplement: Supplementary file 1 — Supplementary [file ADVS-6-1900149-s002.pdf]

Copyright WILEY-VCH Verlag GmbH & Co. KGaA, 69469 Weinheim, Germany, 2019.

ADVANCED SCIENCE Open Access

Supporting Information

for *Adv. Sci.,* DOI: 10.1002/advs.201900149

Investigation of Low-Current Direct Stimulation for Rehabilitation Treatment Related to Muscle Function Loss Using Self-Powered TENG System

*Jiahui Wang, Hao Wang, Tianyiyi He, Borong He, Nitish V. Thakor, and Chengkuo Lee**

Copyright WILEY-VCH Verlag GmbH & Co. KGaA, 69469 Weinheim, Germany, 2019.

Supporting Information

Investigation of Low-current Direct Stimulation for Rehabilitation Treatment Related to Muscle Function Loss Using Self-powered TENG System

*Jiahui Wang, Hao Wang, Borong He, Tianyiyi He, Nitish V. Thakor, Chengkuo Lee**

Supporting Information Table

Figure S1. TENG fabrication process…………………………………………………..…34

Figure S2. Characterization of power output with load resistance of the TENG………35

Figure S3. Characterization of the TENG open-circuit voltage………………………..36

Figure S4. Experiment setup for square wave and enveloped high frequency stimulation testing……………………………………………………………………………………...37

Figure S5. Explanation of motoneuron state change with presence of electrical stimulation………………………………………………………………………………39

Figure S6. Modelling circuit and parameters…………………………………………...41

Figure S7. Quantized result of Figure 7…………………………………………………42

Figure S8. Another set of *in vivo* measurement of motoneuron recruit synchronization…………………………………………………………………………….43

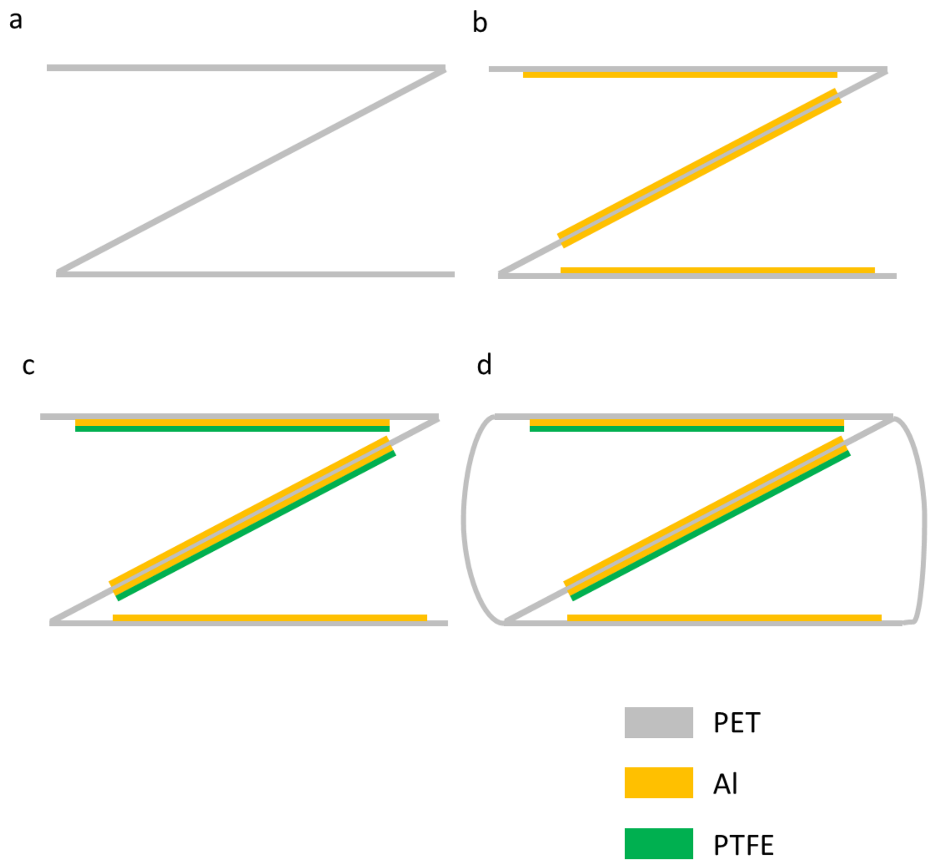

Figure S1. Fabrication process of stacked-layer TENG. (a) PET is folded into zig-zag shape. (b) Aluminum is attached to each side of the PET. (c) PTFE is attached to the top of aluminum. (d) Confinement PET is added.

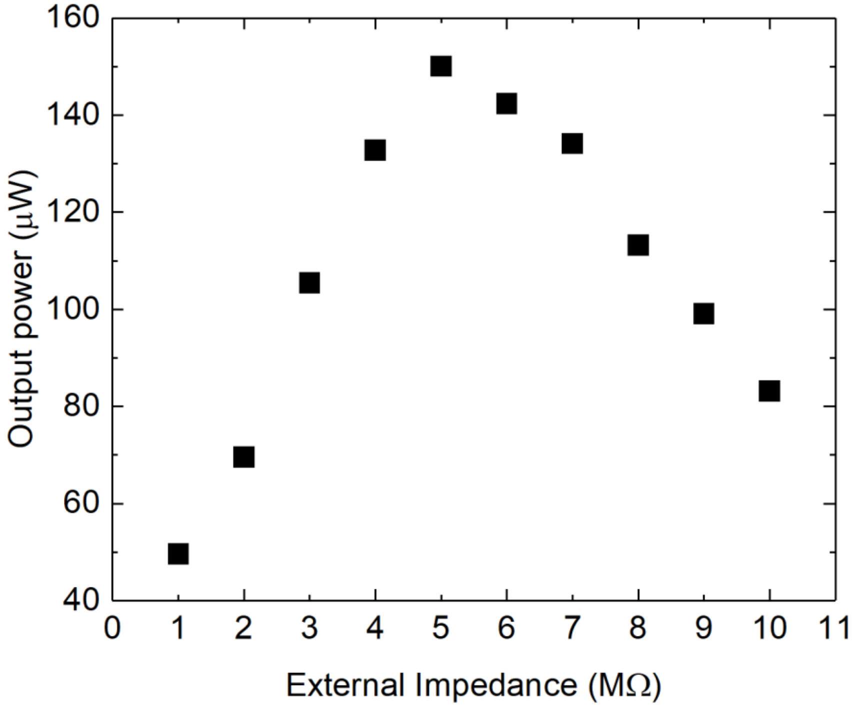

Figure S2. Characterization of power output with load resistance of the TENG.

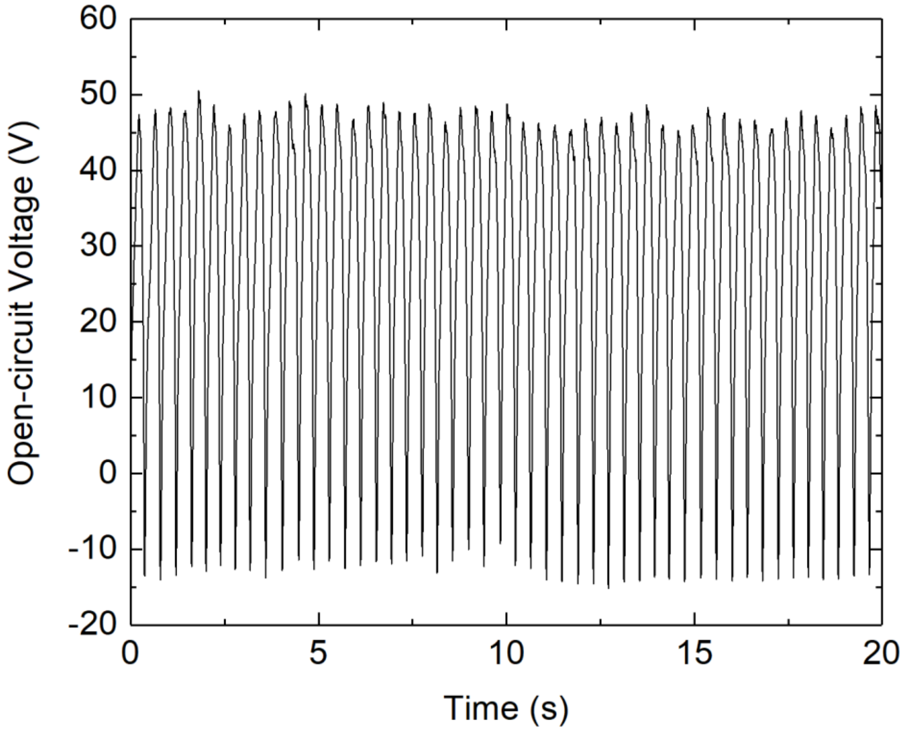

Figure S3. Characterization of the TENG open-circuit voltage.

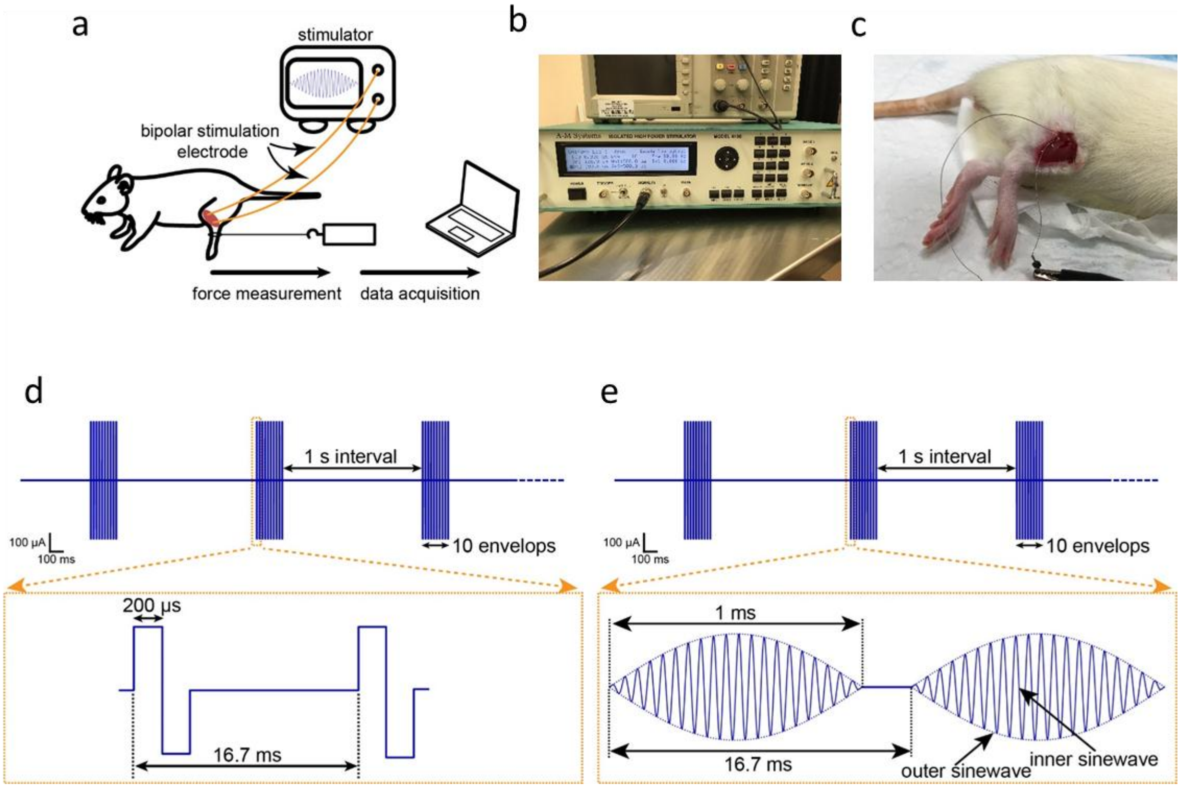

Figure S4. Experiment setup and current stimulation waveform. (a) Experiment setup. Two stainless steel wire electrodes were sutured in the TA muscle. Generated force output was measured by a force gauge connected to the ankle. (b) Photo of the commercial current stimulation. (c) Implantation of the stainless-steel wires. (c) Long pulse width square wave input current. Every 1 s, 10 square wave pulses were delivered. The pulse width of each square wave was 200 μs. (d) Enveloped high frequency stimulation current waveform. Every 1 s, 10 envelops were delivered. Each envelop consisted of gradual ramp-up and gradual ramp-down high frequency pulses.

Motoneuron state change induced by electrical stimulation.

The successful excitation of neurons starts with the generation of APs. In voluntarily controlled muscle movements, one AP sequentially triggers the opening of ion channels on the next node of

Ranvier, so that this AP is relayed along the neuron axon. However, when it comes to electrical stimulation, the transmembrane voltage resulting from electrode-tissue interaction determines the opening of ion channels. Unlike natural APs, this transmembrane voltage not only initiates the opening of ion channels, but also distorts ion distribution by lingering on after the ion channels open. In other words, the long pulse width electrical stimulation still exists after the ion channels open and overlaps with the AP. Figure S3 shows how electrical stimulation distorts ion distribution. During the prior AP, existence of positive electrical stimulation facilitates Na^+ influx and hinders K^+ efflux, resulting in excessive ion influx. In this way, before the subsequent AP, neuron's membrane resting potential ($V_{resting}$) is increased to hover just below the threshold voltage, and the neuron excitability is increased. On the contrary, existence of negative electrical stimulation during the prior AP decreases neuron excitability in the subsequent AP.

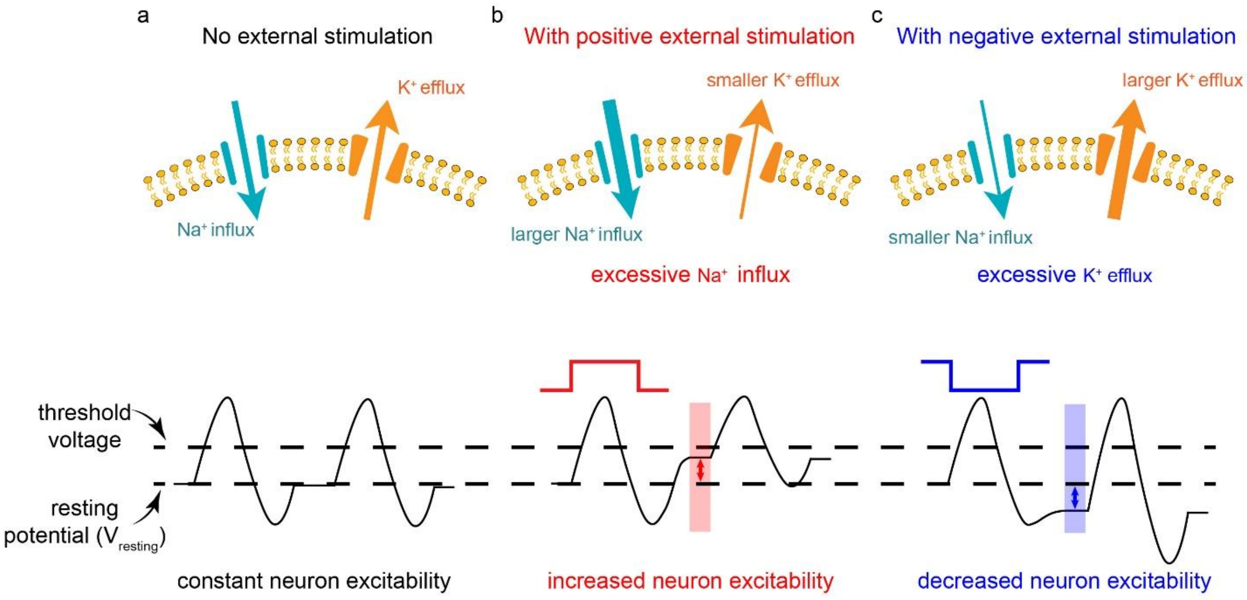

Figure S5. Explanation of motoneuron state change with the presence of electrical stimulation.

Modelling circuit and parameters.

The modelling was performed on MATLAB. Firstly, a circuit description was performed in Simulink (MathWorks, USA). Then, current inputs of different waveforms were recursively fed to the circuit model, and the voltage responses of the targeted RC component were collected (Figure S4a, Figure S4b). In bipolar (two stimulation electrode) electrical stimulation, electric field spreads out in neuromuscular tissue (Figure S4c). Voltage amplitude is larger when closer to the electrodes, and voltage polarity is the opposite at the two electrodes (Figure S4d). Thus, the two stimulation electrodes independently activate nearby neurons and effectively depolarize neuron membrane in different temporal pattern.

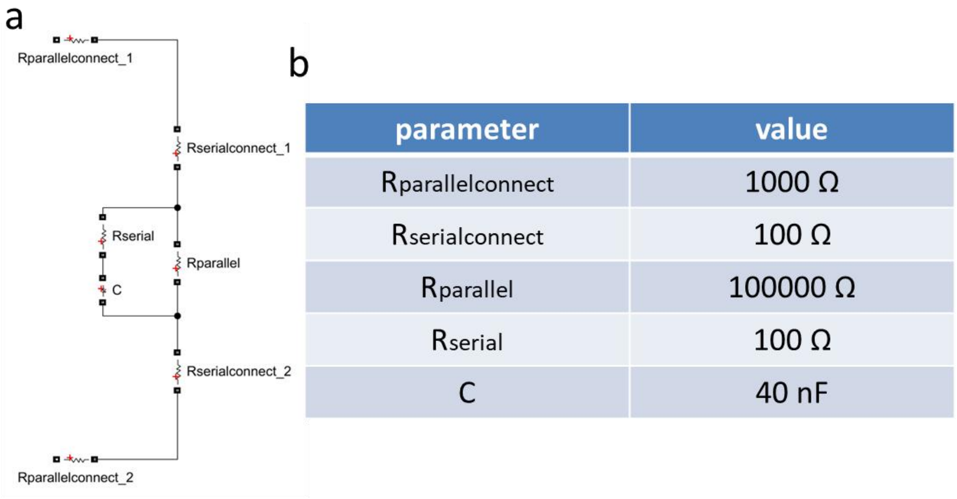

b

parameter	value
Rparallelconnect	1000 Ω
Rserialconnect	100 Ω
Rparallel	100000 Ω
Rserial	100 Ω
C	40 nF

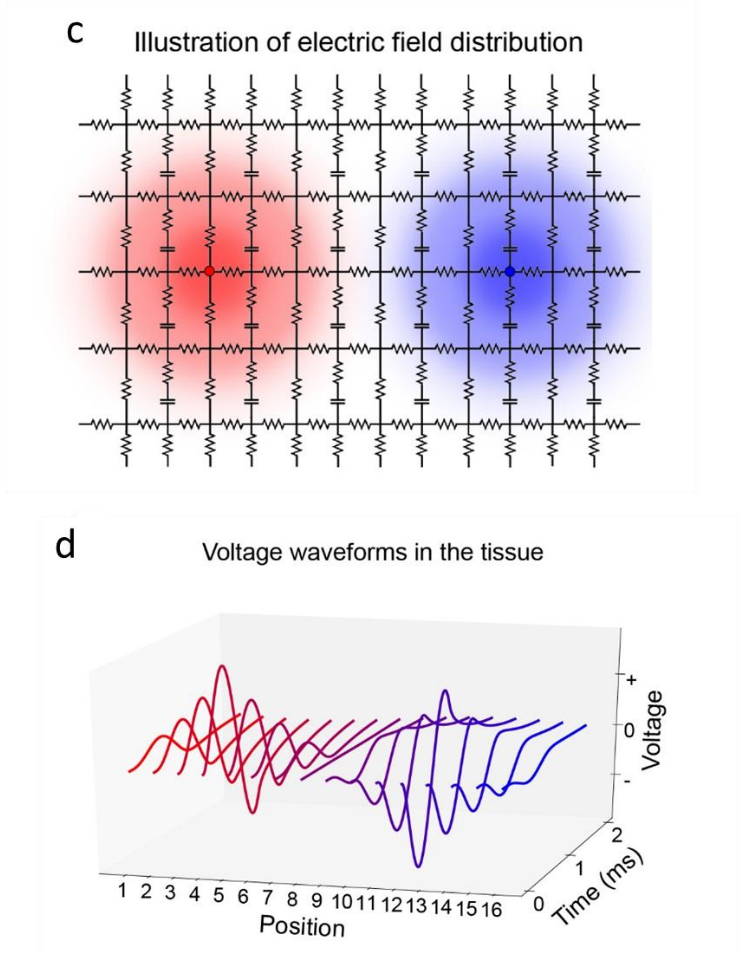

Figure S6. Modelling circuit structure and component parameters.

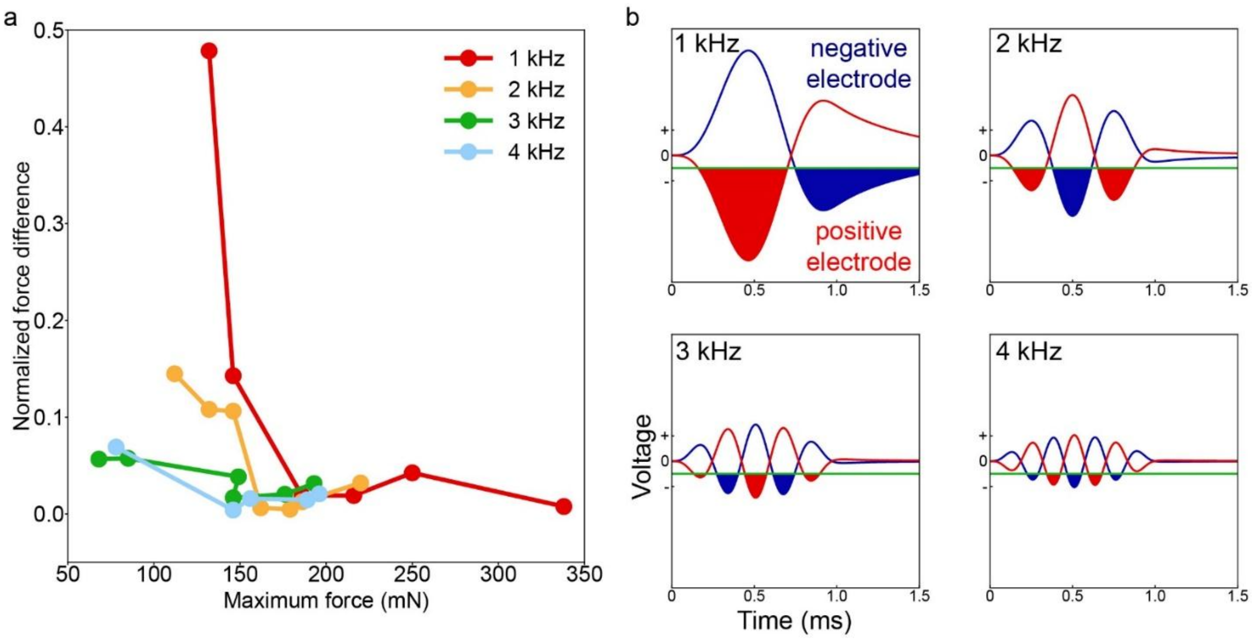

Figure S7. Quantized results of Figure 7. The quantized analysis of the force output difference induced by positive-first and negative-first enveloped high frequency stimulation also confirmed that higher frequency and larger amplitude of stimulation improved neuron recruitment synchronization at the two stimulation electrodes. This can be understood by considering the voltage waveforms at the two stimulation electrodes. At 1 kHz, voltage waveforms exceed threshold voltage in different temporal pattern. With increasing frequency, voltage waveforms at the two stimulation electrodes become synchronized.

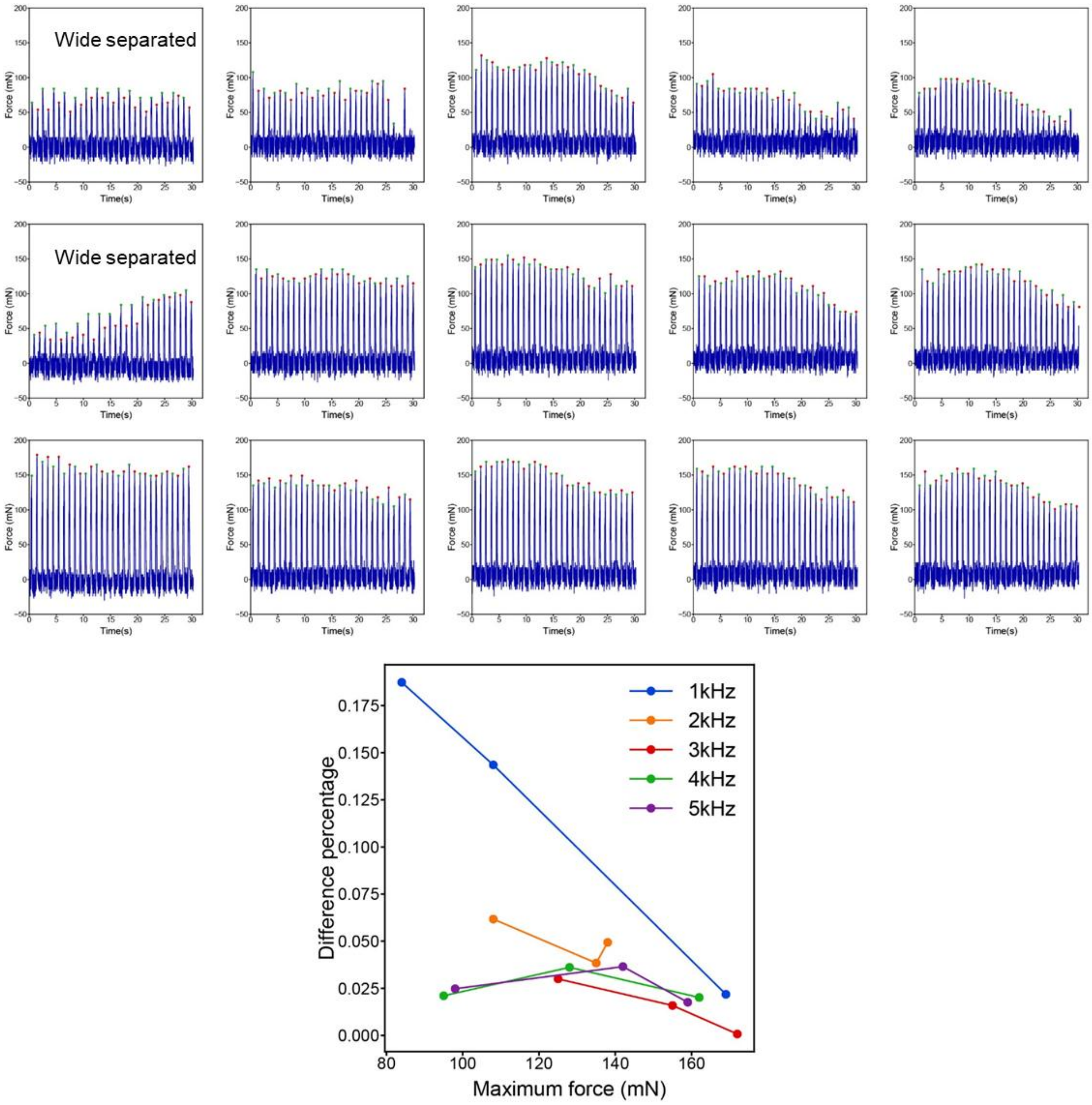

Figure S8. *In vivo* measurements of motoneuron recruitment synchronization on another rat.
